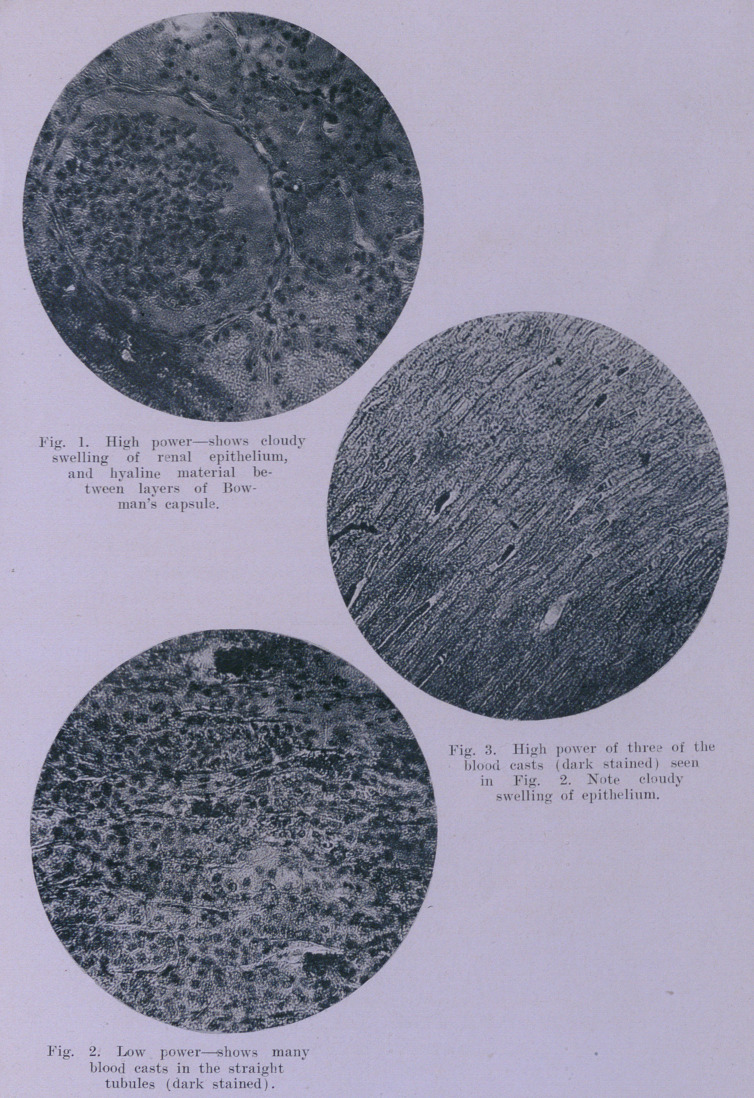# An Unusual Case of Renal Tuberculosis

**Published:** 1916-04

**Authors:** Violet H. Keiller

**Affiliations:** Galveston, Texas


### An Unusual Case of Renal Tuberculosis.

VIOLET H. KEILLER, A. B., M. D., GALVESTON, TEXAS.

The case about to be described occurred in the service of Dr. A. O. Singleton, at the John Sealy Hospital, and it is through his kindness that I am allowed to publish this report. It is remarkable in that the pathological findings seemed to us insufficient to account for the clinical symptoms; and were, moreover, not of the nature that we expect to find in such a condition.

*History:* A. W., female, colored, age 41 years, admitted to John Sealy Hospital May 21, 1915, discharged, improved, July 6, 1915. (Gen. No. 102,226.)

On admission the patient came under the care of the gynecological service, complaining of hematuria of some five months duration, some bladder irritability, and some loss in weight. She had no pain or history of renal colic at this time. Temperature on admission, 99; pulse, 80; respiration, 20. Laboratory examinations made May 22 showed the following:

Urine: Smoky, acid, specific gravity, 1022, heavy sediment, heavy albumen, negative sugar. Microscopic examination: No casts, few leucocytes, many red blood cells.

Blood: Red cells, 3,880,000. Hemoglobin, 76 per cent. White cells, 10,700. Differential count: Polymorphonuclear neutrophiles, 77 per cent; lymphocytes, 19 per cent; large mononuclear leucocytes, 2.5 per cent; transitionals, 1 per cent; eosinophiles, 0.5 per cent.

Cystoscopy on the same day showed healthy bladder, normal except for slight congestion around the right ureteral orifice; ureteral orifices small, contract regularly, easily dilatable. Left jet of urine is bloody and less frequent than the right. Laboratory report on urine from the left ureter was negative for tubercle bacilli.

Patient was transferred to the surgical service, where she came under the care of Dr. A. O. Singleton. On physical examination Dr. Singleton found heart and lungs normal; abdomen apparently normal, except for some tenderness on deep pressure over left kidney. Neither kidney was palpable. The patient at this time was thin, querulous, and complained of constant aching pain in the left renal region. X-ray picture of the kidney was negative. Cystoscopy, June 1, gave same results as the previous examination; urine from each ureter was examined for tubercle bacilli, and a negative report given by the pathologist.

June 2, examination of mixed (bladder) urine showed tubercle bacilli present.

June 4, urine withdrawn from right ureter was negative for tubercle bacilli; urine from left ureter showed tubercle bacilli present.

Throughout this period the patient ran an intermittent type of temperature which was never above 99. She lost weight rather steadily, was nervous, and complained of constant, aching pain in the left renal region. She had no referred pains nor attacks of colic.

*Operation:* On June 6, under ether anesthesia left nephrectomy was performed by Dr. Singleton. The patient made a rather slow but uneventful recovery, leaving the hospital July 6.

*Pathology:* It is to the pathological findings in this case that special interest attaches. On examination of the kidney during operation, the organ was found to be normal in size, capsule normal, no evidence of subcapsular tubercles was apparent, the consistence was apparently normal. In spite of these conditions, removal was thought advisable on the strength of the clinical symptoms and the unequivocal character of the laboratory examinations, which seemed to indicate that nothing short of nephrectomy would give relief. On sectioning the kidney after its removal, the pelvis was found to be reddened, mucosa somewhat thickened, with small erosions from which we judged the bleeding to have come. On naked eye examinations the renal tissue was rather pale but otherwise normal, except for the presence of a single cyst, situated in the medulla, two and one-half centimeters from the upper pole, and measuring approximately one centimeter in diameter, with thin, smooth, slightly sacculated walls, and filled with a creamy fluid. Examination with a low magnification showed the presence of minute areas of hemorrhage throughout the entire kidney.

Microscopic sections were made from many different regions. All showed pronounced cloudy swelling, with occlusion of many tubules by cellular debris; this change was, as might be expected, more noticeable in the convoluted tubules. Faintly staining hyaline material, in a thick layer, was deposited between the layers

Fig. 1. High power—shows cloudy swelling of renal epithelium, and hyaline material between layers of Bowman's capsule.

Fig. 3. High power of three of the blood casts (dark stained) seen in Fig. 2. Note cloudy swelling of epithelium.

Fig. 2. Low power—shows many blood casts in the straight tubules (dark stained).

of Bowman's capsule (i. e., in the very commencement of the renal tubule) surrounding all of the glomeruli. (See Fig. 1.) Some of the glomeruli also showed a round cell infiltration, but no other evidences of such leucocytic infiltrations were found elsewhere in the kidney. Sections through the medulla showed the straight and collecting tubules dilated, filled in many cases with hyaline casts, in other instances with blood casts. (Fig. 2 and 3.) In no section was there evidence of giant cells or of caseation except as the semi-fluid contents of the cyst above mentioned may be interpreted as caseous material. Sections through the pelvis showed some round cell infiltration, slight erosion of the epithelium and some interstitial hemorrhages. An attempt to demonstrate B. tuberculosis in sections of pelvis and of kidney was unsuccessful.

*Post Operative History:* March 18, 1916. Patient has left town, so that personal investigation was impossible. Her sister reports her condition good, and says that she is free from any evidences of disease of lungs or kidneys; that she considers herself cured by the operation, and in good health. She does not think the patient is as strong as she was before the onset of symptoms, fourteen months ago, but except for this slight lack of strength her condition is evidently satisfactory.

## Figures and Tables

**Figure f1:**